# Unraveling the Differences between Gram-Positive and Gram-Negative Probiotics in Modulating Protective Immunity to Enteric Infections

**DOI:** 10.3389/fimmu.2017.00334

**Published:** 2017-03-27

**Authors:** Sukumar Kandasamy, Anastasia N. Vlasova, David D. Fischer, Kuldeep S. Chattha, Lulu Shao, Anand Kumar, Stephanie N. Langel, Abdul Rauf, Huang-Chi Huang, Gireesh Rajashekara, Linda J. Saif

**Affiliations:** ^1^Food Animal Health Research Program (FAHRP), Veterinary Preventive Medicine Department, The Ohio Agricultural Research and Development Center, The Ohio State University, Wooster, OH, USA

**Keywords:** rotavirus, probiotics, *Escherichia coli* Nissle, *Lactobacillus*, immunity, children, diarrhea, gnotobiotic piglet disease model

## Abstract

The role of intestinal microbiota and probiotics in prevention and treatment of infectious diseases, including diarrheal diseases in children and animal models, is increasingly recognized. Intestinal commensals play a major role in development of the immune system in neonates and in shaping host immune responses to pathogens. Lactobacilli spp. and *Escherichia coli* Nissle 1917 are two probiotics that are commonly used in children to treat various medical conditions including human rotavirus diarrhea and inflammatory bowel disease. Although the health benefits of probiotics have been confirmed, the specific effects of these established Gram-positive (G+) and Gram-negative (G−) probiotics in modulating immunity against pathogens and disease are largely undefined. In this review, we discuss the differences between G+ and G− probiotics/commensals in modulating the dynamics of selected infectious diseases and host immunity. These probiotics modulate the pathogenesis of infectious diseases and protective immunity against pathogens in a species- and strain-specific manner. Collectively, it appears that the selected G− probiotic is more effective than the various tested G+ probiotics in enhancing protective immunity against rotavirus in the gnotobiotic piglet model.

## Introduction

Intestinal commensals constitute more than 1,000 species of bacteria. These commensals are involved in nutrient metabolism, development, and functioning of the gastrointestinal (GI) immune system and protection of the host from pathogens (1–3). Colonization of the GI tract is a gradual process in which *Escherichia coli* and other enterobacteria colonize the intestinal tract early after birth, followed by the subsequent establishment of anaerobes (4). The intestinal microbiota of children only becomes adult-like at 2–3 years of age (5). Perturbation of the intestinal microbiota, or dysbiosis, is associated with various diseases such as inflammatory bowel disease (6) and also affects the efficacy of various vaccines in children (7). Probiotics are widely used to restore gut homeostasis in various medical conditions in humans (8–10) and treat diarrheal diseases in children.

Diarrheal disease is one of the leading cause of deaths in children and it accounts for the death an estimated of 700,000 children annually worldwide (11). Specifically, rotavirus (RV) is a major cause of gastroenteritis in children worldwide. The protective efficacy of available RV vaccines is variable between regions and it is lowest in developing countries such as Southern Asia (50.0%) and sub-Saharan Africa (46.1%) (12). Additionally, lack of access to adequate health-care facilities to manage diarrhea is also associated with higher morbidity and mortality in children in low-income settings. Thus, enhancing vaccine efficacy, along with developing economical approaches to reduce the severity of RV diarrhea are effective strategies to ameliorate severe RV disease. Probiotics and intestinal commensals, crucial interacting partners of the gut immune system (13), are increasingly being considered for treatment of various enteric infections including human retrovirus (HRV) diarrhea (14), human norovirus gastroenteritis (15), antibiotic-associated diarrhea (16), and also to modulate protective antiviral immunity (17).

The beneficial effects of probiotics in reducing the severity of RV diarrhea and modulating viral immunity were observed in randomized clinical studies (18) and experimental studies in animal models (19) (Table 1). The Gram-positive (G+) *Lactobacillus* spp. were widely used to treat or prevent RV diarrhea in children. Specifically, prophylactic supplementation of *Lactobacillus rhamnosus* GG (LGG) to children significantly reduced the incidence of HRV disease (20). In our studies, gnotobiotic (Gn) piglets were used to study HRV pathogenesis due to their susceptibility to HRV infection and also the greater anatomic and physiological and immunological similarities between pigs and humans. Dual colonization of Gn piglets with G + LGG and *Bifidobacterium lactis* Bb12 resulted in a significant reduction in both fecal HRV shedding titers and diarrhea severity (21). Further, *Lactobacillus* strains have significant effects in reducing diarrhea severity in children affected by enteric diseases (22).

**Table 1:** **Effects of G+ and G− probiotics on diarrheal diseases and immunity in children and animal models**.

Gram-positive probiotic/commensal bacteria	Gram-negative probiotic/commensal bacteria	Humans/animal model/*in vitro* study	Indication	Conclusion(s)	Reference
*L. rhamnosus* GG (6 × 10^9^ CFU/dose)	None	Children	Prophylaxis against diarrheal diseases	Significant reduction in incidence of HRV disease in LGG-supplemented group	(20)

*L. rhamnosus* GG (10^10–11^ CFU)	None	Children	Effect of LGG on immune responses to HRV in children	LGG significantly enhanced RV-specific IgA antibody responses	(23)

*L. rhamnosus* GG (10^10^ CFU)	None	Children	Treating diarrhea	Reduced duration of diarrhea	(24)

*Lactobacillus paracasei* strain ST11 (10^10^ CFU)	None	Children	Treating diarrhea	Reduced severity of non-rotavirus induced diarrhea but no effect on rotavirus diarrhea	(25)

None	*Escherichia coli* Nissle 1917 (EcN) (10^8^ CFU)	Children	Treat acute diarrhea in children	Reduced duration of diarrhea by 2.3 days	(26)

None	EcN (3 × 10^8^ CFU)	Infants	To assess effects on total IgA responses in infants	Increased serum and stool IgA responses	(27)

None	EcN (10^8^ CFU)	Infants	Assess impact on cellular and humoral immunity in infants	Probiotic increased both cellular proliferative and serum total IgA responses	(28)

None	EcN (10^8^ CFU)	Infants	Prophylactic administration against bacterial pathogens	Significant reduction in bacterial pathogens in fecal samples	(29)

*Bifidobacterium choerinum* (5 × 10^8^ CFU/ml)	EcN (5 × 10^8^ CFU/ml)	Gn piglets	Protection against *Salmonella enterica* serovar Typhimurium infection	EcN conferred higher protection against disease than *Bifidobacterium choerinum*	(30)

*L. rhamnosus* GG (10^5^ CFU/ml)	EcN (10^5^ CFU/ml)	Gn piglets	Compare G+ and G− bacteria effect on HRV infection and immunity	EcN was more effective than LGG in ameliorating HRV disease and enhancing total IgA and NK cell responses	(31, 32)

*L. rhamnosus* GG (10^5^ CFU/ml), *Bifidobacterium lactis* Bb12 (105 CFU/ml)	None	Gn piglets	To study effects on HRV disease	Reduced fecal virus shedding and diarrhea severity in probiotic colonized piglets	(21)

*Enterococcus faecium* NCIMB 10415 (4.2–4.3 × 10^6^/g CFU)	None	Sows and their offspring	Effect on fecal shedding of enteric viruses	Reduced fecal shedding of rotavirus and increased rotavirus specific IgA responses. No effect on hepatitis E virus, encephalomyocarditis virus, and norovirus shedding in feces	(33)

None	EcN (10^10^ CFU/ml)	Pigs	To prevent enterotoxigenic *Escherichia coli* induced diarrhea	Ameliorated clinical signs of diarrhea	(34)

None	EcN (10^8^ CFU/ml)	Neonatal calf	Prevention and treatment of diarrhea	Reduction in incidence of diarrheal diseases in prophylactic group. Ameliorated severity of diarrhea in calves with enteric diseases	(35)

*Lactobacillus acidophilus* A9 (10^8^/ml CFU)	*Escherichia coli* 13-7 (10^6^/ml CFU)	Mice	Compare G+ and G− bacteria effect on cytokine responses in mice	*E. coli* 13-7 induced higher IL-12 cytokine compared to *L. acidophilus* A9	(36)

None	EcN (1.5–2 × 10^8^ CFU)	Mice	Assess impact on intestinal barrier function in acute dextran sodium sulfate-induced colitis	Strengthened intestinal barrier function	(37)

*Lactobacillus casei* Shirota	EcN	*In vitro*	Investigate effects on innate immunity	Higher IL-10 and IL-12 induction by EcN than *L. casei* Shirota	(38)

*L. plantarum, L. rhamnosus, L. paracasei* ssp. paracasei	*Escherichia coli* O6:K13:H1, *Escherichia coli* MS101	*In vitro*	Compare G+ and G− bacteria effect on cytokine responses of monocytes	Lactobacilli-induced higher level of IL-12	(39)

The effectiveness of probiotics in preventing or treating a disease is dependent on several factors such as class or strains of probiotics, the dosage of probiotics, and heterogeneity of study subjects (40, 41). Several past studies showed strain-specific differences of probiotics in modulating host immune responses (42). Thus, comparative analysis of the health benefits of different classes of probiotics is essential to tailor an effective regimen of probiotic treatment for a disease condition. Specifically variations in microbe-associated molecular patterns between G+ and Gram-negative (G−) bacteria have been attributed to differential induction of innate immunity in a host (43, 44). However, limited studies have been conducted to decipher if differences exist between G+ and G− probiotics in modulating host responses to infectious diseases. In our recent studies (31, 32), we compared the beneficial effects of G+ and G− probiotics in modulating virulent HRV infection as well as host immunity. Specifically, LGG was selected as a G+ probiotic because of its well-documented effects in reducing the severity of RV diarrhea in children (24). For the G− probiotic, we selected *Escherichia coli* Nissle 1917 (EcN) due to its proven effects in attenuating inflammatory disorders and modulating immunity in humans (45). In this review, we focused on the comparisons of the health benefits of G+ and G− probiotics in modulating microbial infections and immunity.

## Effects of G+ versus G− Probiotics on Enteric Infections and Diarrhea

Probiotics have been successfully used to prevent or treat enteric infections in children and animals (Table 1). One notable finding is the difference between G+ and G− probiotics in modulating host immunity against microbial diseases. In one study (31), the comparative efficacy of LGG and EcN probiotics in ameliorating HRV disease was assessed in Gn piglets. The EcN colonized piglets had reduced diarrhea severity and also lower mean peak virus shedding titers compared with LGG or uncolonized piglets post-virulent human RV (VirHRV) challenge (31, 32). Both EcN and LGG showed similar colonization patterns as indicated by comparable fecal shedding of each bacterium and also detection of similar levels of each probiotic bacteria in various sections of GI tract. Similarly, EcN supplementation to children with enteric infections resulted in reduced duration of diarrhea (26). Further, supplementation of EcN to infants for the first 5 days immediately after birth resulted in persistence of the probiotic for 6 months as indicated by fecal shedding of EcN (29). Similar to the higher beneficial effects of EcN than LGG on ameliorating HRV infection, higher protective effects against *Salmonella* were observed in EcN compared with *Bifidobacterium choerinum*-supplemented Gn piglets (30). The higher protective effect of EcN against *Salmonella* was associated with increased expression of ZO-1 and occludin in ileal epithelial cells and decreased inflammatory TNF-α cytokine levels in the EcN colonized Gn piglets (30). Consistent with these findings, higher TNF-α levels were induced by G+ commensals as compared with G− commensals using *in vitro* mononuclear cultures (43). EcN supplementation also attenuated lipopolysaccharides (LPS) or trinitrobenzene sulfonic acid-induced inflammatory conditions in a mouse model (46). In summary, the higher ability of G− compared with G+ probiotics in reducing the levels of inflammatory mediators during enteric infections may be major contributing factor in reducing diarrhea severity.

### G+ and G− Probiotic Impacts on Modulation of B Cell Responses

**Figure 1:**
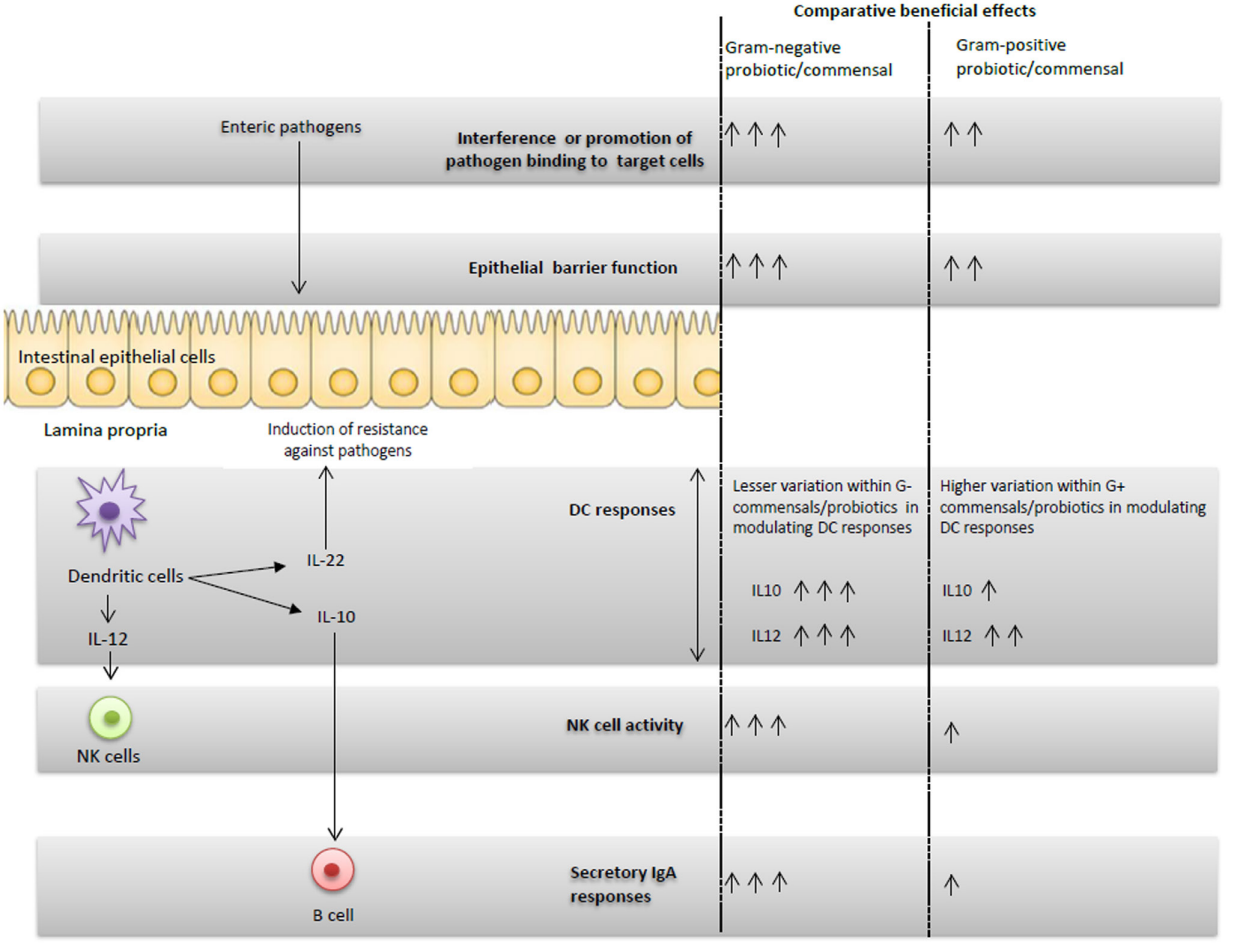
**Schematic representation of the G+ and G− probiotics-induced immunomodulatory effects and proposed potential immune interactions**.

Microbial colonization of the GI tract has a significant effect on the maturation of neonatal immune system (47). Consistent with this observation, administration of EcN enhanced serum EcN-specific IgA antibody and polyclonal IgM antibody responses in infants as compared with placebo group (28). Also, mono EcN or dual EcN + LGG colonization significantly increased serum total IgA and IgG responses compared with LGG colonized or uncolonized piglets (31) (Figure 1). Similar to systemic immunoglobulin responses, EcN colonization resulted in higher small intestinal total IgA responses compared with LGG colonization in Gn pigs. Thus, EcN had more potent immunostimulatory effects than LGG in terms of inducing mucosal and systemic B cell responses. The underlying mechanism for differential induction of antibody responses by G+ and G− bacteria might be due to variation in IgA inducing factors such as IL-10 cytokine. In fact, G−, but not G+ probiotics, induced higher IL-10 responses in prior studies (31, 48, 49). IL-10 is one of the cytokines that mediates the induction of IgA antibody responses at mucosal sites through enhancing antibody class switching (50). Differences in the microbe-associated molecular patterns between the probiotics might be a potential reason for the differential induction of IL-10 by G+ and G− bacteria. Indeed, both the LPS portion of EcN and whole EcN lysate were identified as potent inducers of IL-10 production in peripheral blood mononuclear cells (51). Further, induction of total IgA responses is at least partially mediated by IL-10 *in vitro* (31). These studies demonstrate that modulation of the cytokine milieu, such as enhanced IL-10 levels, might be a potential mechanism to account for the higher antibody responses observed in G− compared with G+ probiotics groups.

It is also well established that strain-dependent variations in immunomodulatory properties are observed within G+ probiotics (52). Thus, individual probiotic strains within G+ or G− probiotic classes may differ in modulating antibody responses. Consequently, screening of the beneficial effects of individual probiotics is essential to elucidate their impacts on antibody responses.

### Impact of Innate Immunity on IgA Responses to G+ versus G− Probiotics

Innate immunity plays an integral role in priming the adaptive immune responses. Thus, probiotics may induce specific changes in innate immunity that may be involved in synergistically enhancing IgA responses. Dual colonization of a G− and G+ probiotic enhanced serum total IgA responses in Gn piglets compared with mono-colonization of the probiotics (31). Thus, G+ and G− bacteria synergistically enhanced the systemic total IgA responses. In fact, combinations of G+ and G− probiotics had additive effects on induction of maturation markers in DCs as well as levels of IL-10 cytokines (53). Thus, considering the known function of DCs in induction of IgA responses (54, 55), the positive effects of combinations of G+ and G− bacteria in modulating DCs may play a role in enhancing IgA responses. Additionally, a previous study (56) also showed that LPS, a TLR4 ligand, synergistically interacted with TLR1/2 ligands which in turn enhanced class-switch recombination in B cells. Thus, synergistic interactions of microbe-associated molecular patterns from G− and G+ probiotics might also play a role in enhancing antibody responses. Apart from DCs, it appears that intestinal epithelial cells also respond differently in terms of producing IgA mediators such as TGF-β and thymic stromal lymphopoietin (TSLP). Specifically, G− commensals induce higher production of TGF-β and TSLP as compared with G+ commensals (57). Further, higher frequencies of splenic TLR9^+^ mononuclear cells were detected in EcN + LGG colonized compared with the monocolonized EcN or LGG Gn piglets (32). TLR9 recognizes CpG DNA and LGG has a high GC percentage in its genomic DNA (58). Thus, we speculate that higher systemic TLR9 expression in EcN + LGG compared with EcN or LGG monocolonized piglets might be a contributing factor in enhancing immunoglobulin responses as reported in several earlier studies (59, 60).

One unanswered question is the involvement of total IgA levels in modulating immunopathology during microbial infections. Previous studies have shown the involvement of IgA in moderating inflammatory responses through modulating dendritic cells and regulatory T cell functions (61, 62). Further, secretory IgA-commensal complexes were shown to reduce inflammatory responses in intestinal epithelial cells (63). Thus, the role of secretory IgA in mitigating infection-induced inflammatory responses is intriguing and requires further investigation.

### Differential Effects of G+ versus G− Probiotics in Modulating Innate Immunity

Probiotics may elicit their beneficial effects against pathogens through modulating innate immunity. A role for innate immunity in mediating host defenses against enteric diseases including RV infection has been elucidated in recent studies (64–67). Specifically, functions of dendritic cells are modulated by various probiotics. It appears that DC populations in the intestine can be modulated by intestinal commensals. This concept is supported by results of an investigation in which depletion of intestinal microbiota resulted in a reduction in DCs numbers in mucosal compartments as well as impaired resistance against influenza virus infection in mice (68). Additionally, G− commensal bacteria have higher immunostimulatory effects on DCs as compared with G+ commensals (69). For example, G− EcN increased frequencies of total plasmacytoid dendritic cells (pDCs) and activated pDCs, more than the G+ LGG probiotic in Gn piglets (32). Also, G− commensals were highly potent in the induction of maturation markers in DCs as compared with G+ commensals (53). Importantly, greater variation was observed among G+ commensals in modulating DC responses, compared with less variation among G− commensals (53). Thus, the distinct ability of G− bacteria such as EcN in modulating frequencies and functions of DCs may have beneficial impacts on induction of protective immunity against pathogens.

In our recently published study (32), we observed higher NK cytotoxic function and increased frequencies of pDCs in EcN colonized compared with LGG colonized or uncolonized piglets. The enhanced NK cell activity coincided with higher serum IL-12 levels *in vivo* in EcN colonized piglets (Figure 1) and also DC production of IL-12 *in vitro* (32). Similar to our studies, treatment of murine bone marrow-derived DCs (BMDCs) with EcN resulted in induction of IL-12 and IL-10 cytokines and induction of activation markers in BMDCs (70). In the same study, EcN administration reduced the development of allergen-specific Th2 responses (70). Thus, our results showed that NK cell function can be modulated by probiotics, and more importantly, only G− EcN but not G+ LGG, enhanced NK cell function. These findings were further corroborated by an earlier study in which the germ-free condition impaired the priming of NK cell function by microbial ligands (71). Further, the reduced NK cell function in microbiota-depleted mice was correlated with higher mouse cytomegalovirus titers post-viral challenge (71). A recent study (72) also showed the potential role of the outer membrane vesicles from EcN in induction of IL-22 cytokine responses. IL-22, along with IFN-λ, has been shown to effectively reduce RV replication in a mouse model (66). These results underscore not only the importance of intestinal commensals in regulating innate immunity against viral infections, but also the differential abilities of distinct known G+ or G− probiotics in regulating innate immune cells.

### Interactions between Commensals and Viruses That Alter Their Pathogenesis

Direct interactions between viruses and bacteria are being increasingly investigated in recent studies (73–75). Specifically, direct binding of commensal microbiota is associated with either increased or decreased viral infections (76). The ability of mouse mammary tumor virus to bind with LPS was associated with increased virus pathogenicity (77). Similarly, poliovirus stability and viral attachment to target host cells were also enhanced by interaction with bacterial LPS or peptidoglycan (78). Further, EcN binds to HRV *ex vivo* but no such interaction was found between LGG and HRV (31). Also, in this study, prior treatment of epithelial cells with EcN, but not LGG, resulted in a significant reduction in the epithelial attachment of HRV *in vitro*. Further studies are required to elucidate the potential role of physical interactions between EcN and viruses in terms of altering the course of viral infection and pathogenicity. Expression of histo-blood group antigens (HBGA) was observed in some G− intestinal commensal bacteria (79) and certain of those HBGA-expressing bacteria were shown to enhance (73) enteric viral infection. Considering the direct interactions between the commensals and pathogens, any disturbances in microbiota compositions may lead to altered susceptibility or resistance to a particular enteric pathogen. Thus, further studies are required to assess whether any difference exists between G+ and G− bacteria in binding properties with various enteric viruses and the impact on the course of viral pathogenicity.

## Conclusion

Comparison of the beneficial effects of G+ and G− probiotics and intestinal commensals indicated that the selected G− probiotic had higher beneficial effects in inducing protective immunity against enteric pathogens such as HRV as compared with the selected G+ probiotics in humans and animal models. In our simplified *in vivo* Gn piglet model system, it appears that the induced beneficial effects of G− EcN against HRV disease may be accomplished by the integrated interaction of DCs, NK cells, and immunoglobulins as well as direct binding of EcN to virus (Figure 1). Most of the initial studies showed that G− probiotics have higher immunostimulatory effects and better protective effects against HRV as compared with G+ probiotics. It remains to be determined whether these findings can be generalized to all G− commensals. Further, the potential ability of different G+ and G− probiotics to alter the composition as well as functionalities of the intestinal microbiota, and the consequences of these changes on microbial infections and vaccines is unclear. Identification of the essential components of probiotics that induce the beneficial effects against pathogens may also be useful in identifying probiotics or their products as novel adjuvants for vaccines.

## Author Contributions

All authors listed have made substantial direct contribution to the work.

## Conflict of Interest Statement

The authors declare that the research was conducted in the absence of any commercial or financial relationships that could be construed as a potential conflict of interest.
